# Postmortem interval estimation of time since death: impact of non-histone binding proteins, immunohistochemical, and histopathological changes in vivo

**DOI:** 10.25122/jml-2024-0260

**Published:** 2024-09

**Authors:** Abdullah Mohammed Karamallah Albloshi

**Affiliations:** 1 Anatomy Department, Faculty of Medicine, Al-Baha University, Al-Baha, KSA

**Keywords:** postmortem interval, gastrocnemius muscle, HMGB1, desmin

## Abstract

The postmortem interval (PMI) is one of the primary objectives and challenging tasks proposed for determining the time of death. This study aimed to estimate the PMI using serum levels of high mobility group box 1 (HMGB1), a biomarker of pyroptotic cell death, along with desmin immunohistochemical and histological analyses of the gastrocnemius muscle in rats at various time intervals. Serum and gastrocnemius muscle samples were collected at zero, 24-, 48-, 72-, and 96 hours postmortem from 50 rats maintained at 22 ± 2°C. The results revealed that the HMGB1 level peaked at 48 hours and dropped in a time-dependent manner afterward. Immunohistochemical analysis revealed a progressive decrease in desmin expression, with severe immunoreactivity (38.19%) at 0 hours, dropping to a minimal level (1.09%) 96 hours after death. Histological analysis of the gastrocnemius muscle at 96 hours revealed significant vacuolation, loss of normal architecture, reduced nuclear visibility, and complete autolysis of all myocytes. In conclusion, HMGB1 levels, desmin immunoreactivity, and histopathological alterations seen in the gastrocnemius muscle could be helpful, valuable, and potential markers for accurately determining PMIs in humans in future studies.

## INTRODUCTION

The human body undergoes complicated changes after death, which are known to be influenced by internal and external variables. Both the environment and the way an individual dies can have a substantial impact on the rate of decomposition. Nevertheless, it has been shown that the decomposition process is predictable, providing the opportunity to determine the postmortem interval (PMI), or the duration since death, based on microscopic and/or gross morphological changes to the body. A precise and accurate PMI estimation can assist medicolegal investigators in identifying the deceased, help establish the chronology of events leading up to death, and confirm or refute other forensic evidence [1].

Determining the PMI is one of the primary objectives and most challenging tasks of forensic pathology. Even though this parameter is unquestionably vital, particularly in criminal investigations, many studies on the subject only address the postmortem parameters' time dependency, which has a minimal bearing on actual forensic practice [2]. Contempt for the emergence of multiple novel methods for PMI assessment in recent years [3] indicates that methods based on the examination of rigor, algor, and livor mortis continue to be the most commonly utilized. However, a variety of individual and environmental factors, such as age, gender, ambient temperature, and physiological and pathological states, can have an impact on these variables. They are primarily the result of physical and chemical processes during the postmortem period. Because of this, traditional methods frequently exhibit limitations in their application, as well as inaccuracy and unreliability [4]. All body tissues undergo a series of extensive biochemical alterations following death due to low oxygen levels, hampered enzymatic reactions, lack of metabolites, and disintegration of cellular components. These biochemical alterations may serve as indicators for a more precise PMI assessment [5].

Researchers have looked into biochemical markers that aid in determining the amount of time that has passed since death. These consist of protein fractions, urea, creatinine, glucose, iron, potassium, calcium, enzymes, myo-albumin fraction, and the amount of strontium-90 calcium analogs [6]. Another one of these is the immunohistochemical detection of insulin in pancreatic β-cells. Medical procedures like measuring rigor mortis, checking the liver, or taking a body temperature can only be used to accurately measure the PMI in the first two or three days after death [7]. There is also a negative correlation between the significance and practical utility of PMI estimation and the amount of research focused on it [8].

One of the most critical areas of research in PMI estimation involves the protein high mobility group box 1 (HMGB1), a nuclear protein with a highly conserved amino acid sequence across species found in many eukaryotic cells. HMGB1 seems to have two distinct functions in cellular systems. First, it acts as an intracellular transcription regulator, essential for maintaining DNA function. Second, during necrosis, triggered by factors such as lipopolysaccharides, tumor necrosis factor-alpha, interleukin-1, and interferon-gamma, HMGB1 translocates from the nucleus to the periphery and is released from macrophages [9].

In this study, gastrocnemius muscle tissue was used because it undergoes autolytic changes and putrefaction more slowly than body fluids and other organs. Despite limited studies on the role of HMGB1 in PMI estimation, its involvement is promising. As necrosis is part of the postmortem process, and necrotic cells release HMGB1, detecting serum HMGB1 from necrotic tissue could provide a potential link to PMI [10].

This research aimed to examine the potential of serum HMGB1 as a postmortem marker using ELISA analysis in 50 male Wistar rats. Additionally, immunohistochemical staining of desmin, combined with histopathological changes in the gastrocnemius muscle, was evaluated to assess the effects of these biomarkers in estimating PMI.

## Material and Methods

### Animals

All methods were conducted in accordance with ARRIVE (Animal Research: Reporting of In Vivo Experiments) guidelines and regulations. Male Wistar rats were purchased from King Abdulaziz University in Jeddah, Saudi Arabia. Before beginning the experiment, the animals were given one week to acclimate to the laboratory environment. They were kept in metal cages for the experiment, provided a standard diet, and had unlimited access to water. The rats were kept in a 12-hour light-dark cycle with a controlled temperature of 22–24°C and relative humidity of 50–60% at the Anatomy Department, Faculty of Medicine, Al-Baha University.

### Experimental animals and animal grouping

Fifty male Wistar rats weighing 230 ± 10 g and 12 weeks old were used in this study. The rats were divided into five groups of ten and housed in plastic cages with free access to water and a standard laboratory diet. After light sedation with halothane, the rats were sacrificed by cervical dislocation. Skeletal muscle samples were collected at 0, 24-, 48-, 72-, and 96-hours postmortem. The first group, serving as the control, had samples taken immediately after sacrifice (0 hours). In contrast, samples were taken at 24-, 48-, 72-, and 96-hours postmortem for the second, third, fourth, and fifth groups, respectively. The sacrificed rats were maintained at room temperature throughout the experiment, 22 ± 2ºC during the day and 9 ± 2ºC at night.

Rats were chosen as the model for this study due to their physiological and morphological similarities to humans. Additionally, the gastrocnemius muscle is easier to extract in rats compared to mice. The use of rats for experimentation was conducted in strict accordance with ethical standards and the internationally recognized guidelines for the care and use of laboratory animals (Approval Ethical Number: REC/ANT/BU-FM/2024/55).

### Rat samples for HMGB1 ELISA detection

Blood samples were obtained from the heart and the great vessels during the autopsy. The samples were centrifuged at 3,000 rpm for 5 minutes and stored at -80°C for further analysis. Serum HMGB1 concentration was determined with an ELISA kit (ThermoFisher; Sci, Cat no: EEL102) [11].

### Immunohistochemical and histological studies

Tissue samples were fixed in 10% formal saline for 48 hours, rinsed with tap water, and processed to create paraffin sections for immunohistochemical and histological analysis. The skeletal muscle sections used in the immunohistochemical study were mounted on charged slides, deparaffinized with two fresh xylene changes, and rehydrated in graded ethanol (99%, 95%, and 70%) before being rinsed in PBS for ten minutes. Antigen retrieval was then carried out after the endogenous peroxidase activity was inhibited. Specimens were then kept in an incubator at room temperature. The primary desmin antibodies (Sigma Aldrich Company) were applied to the sections and incubated for the entire night at room temperature in a humidified chamber. Each section received a labeling antibody, and dimethoxybenzidine was used as a chromogen. After that, the sections were mounted in DPX, counterstained, and dehydrated in increasing alcohol grades before being inspected under a regular light microscope [12]. For histological analysis, gastrocnemius muscle samples were embedded in paraffin blocks, cleared with xylene, dehydrated with alcohol, and fixed in buffered formalin. Hematoxylin and eosin stain were applied after the paraffin blocks were cut into 5 µm thickness [13].

### Sample size

The sample size was determined based on similar previous studies [14]. Ten samples were determined in each group using G*power v.3.1.9.5, which was used to calculate the sample size based on an effect size of 1.6254, a two-tailed test, an α error of 0.05, and a power of 90.0%.

### Computer-assisted digital image analysis (digital morphometric study)

Slides were photographed using an MVV5000CL digital eyepiece installed on a MEIJI MX5200L microscope and Future WinJoe software using a 200X objective. An Intel Core I7-based computer was used to analyze the resulting 20X images for immunohistochemical staining surface area percentage. To achieve this, Fiji ImageJ (version 1.51r; NIH) software was used, along with the color deconvolution 2 plugin (histological dyes digital separation). As a result, three separate digital images were produced (hematoxylin, DAB, and supplementary). The threshold function was used on the DAB channel in immunohistochemistry for intensity calibration, followed by positive area percentage measurement. Data was exported to Excel for further analysis. Five random fields sized 200×200 µm from each slide were analyzed and averaged. In order to determine the histological autolysis score, samples were analyzed and ranked using a semi-quantitative scale in relation to the control samples: 1 denotes minimal autolysis (<5%), 2 mild autolysis (5–10%), 3 moderate autolysis (10–50%), and 4 severe autolysis (>50%). For every slide, a minimum of ten fields (10X) were examined and averaged [15].

### Statistical analysis and data interpretation

GraphPad Prism 8 (GraphPad Software) was used to analyze the data. The Kolmogorov-Smirnov and Shapiro-Wilk tests were used to assess the normality of numerical data. The surface area percentage data from immunohistochemical staining displayed a normal (parametric) distribution. The mean and standard deviation (SD) values of the data were displayed. The groups were compared using a one-way ANOVA and an ad hoc Tukey's multiple comparison test. The significance level of these results was determined at 0.05.

## Results

### Postmortem change of serum HMGB1 levels

**Figure 1:**
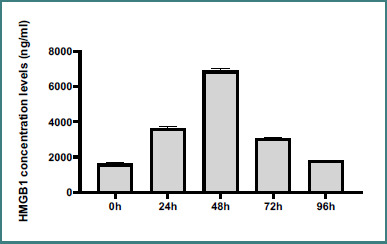
Time-dependent changes in HMGB1 concentration in the serum of rats postmortem

Serum HMGB1 levels were measured during the postmortem period using ELISA. The study found that serum HMGB1 levels in the deceased rats at 22 ± 2°C had significant changes over time. HMGB1 levels peaked on the second-day postmortem, followed by a gradual decline over the third and fourth days in a time-dependent manner (Table 1, Figure 1).

**Table 1:** Time-dependent changes in HMGB1 concentration levels (ng/ml) in the serum of rats postmortem

Group	0h	24h	48h	72h	96h	*P* value
**HMGB1 concentration levels (ng/ml) Mean**	**1609**	**3642.3^a^**	**6890.6^ab^**	**3067.1^abc^**	**1788^abcd^**	**<0.0001F = 6675**
**SD**	**± 71.87**	**± 77.96**	**± 135.46**	**± 56.15**	**± 35.50**	

Values expressed as mean ± SD. Statistical analysis was conducted using one-way ANOVA followed by Tukey's post-hoc multiple comparison test. a: Significance vs. 0h; b: Significance vs. 24h; c: Significance vs. 48h; d: Significance vs. 72h at *P* < 0.05.

### Immunohistochemical findings: cleaved desmin expression

**Figure 2:**
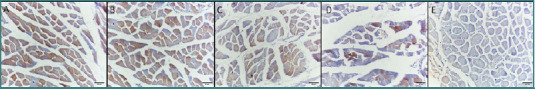
Desmin immunohistochemical staining micrographs of gastrocnemius muscle sections at 200X magnification. A, Gastrocnemius muscle at zero hours, showing severe immunoreactivity of almost all the muscle fibers; B, Gastrocnemius muscle at 24 hours, showing moderate immunoreactivity of the muscle fibers; C, Gastrocnemius muscle at 48 hours, showing mild immunoreactivity of the muscle fibers; D, Gastrocnemius muscle at 72 hours, showing minimal immunoreactivity of the muscle fibers; E, Gastrocnemius muscle at 96 hours, showing minimal to no immunoreactivity of the muscle fibers.

When the PMI increased, positively stained areas gradually decreased, according to desmin immunohistochemical staining. The greatest increase in desmin expression (nuclear and cytoplasmic) occurred at 0 hours postmortem. After 24 hours, moderate to high cytoplasmic desmin expression was found, followed by moderate expression 48 hours later (72 hours from time of death), with weak expression occurring 96 hours from time of death (Figure 2A-E, Table 2).

**Table 2:** The quantified comparison of immunohistochemical staining desmin in the different rat groups versus time intervals

Group	0h	24h	48h	72h	96h	*P* value
**Desmin muscle staining area percentage Mean**	**38.19**	**25.89^a^**	**12.54^ab^**	**7.07^abc^**	**1.09^abcd^**	**<0.0001 F = 55.72**
**SD**	**± 7.27**	**± 11.64**	**± 1.83**	**± 3.18**	**± 0.28**	

Values expressed as mean ± SD. One way ANOVA followed by Tukey's post-hoc multiple comparison test. a: Significance vs. 0h, b: Significance vs. 24h, c: Significance vs. 48h, d: Significance vs. 72h at *P* < 0.05.

### Postmortem gastrocnemius muscle histological findings

**Figure 3:**
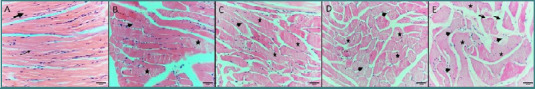
Hematoxylin and eosin (H&E) stained micrographs of skeletal muscle sections. **A**, skeletal muscle at zero hours, showing normal myocytes with peripheral placed spindle nuclei and cross striation; **B**, Skeletal muscle at 24 hours, showing minimal autolysis of myocytes and the appearance of rounded nuclei; **C**, Skeletal muscle at 48 hours, showing moderate myocytes autolysis and increase in the rounded nuclei; **D**, Skeletal muscle at 72 hours, showing extensive autolysis and necrosis of myocytes with round or disappeared nuclei; **E**, Skeletal muscle at 96 hours, showing complete autolysis of all myocytes, reduced visible nuclei number, and vacuolation with loss of normal architecture. Thick arrow: myocytes, thin arrow: spindle peripheral myocytes nuclei, arrowhead: round nuclei, star: myocytes autolysis, curved arrow: vacuolation and loss of architecture. 200X magnification.

Hematoxylin and eosin-stained sections of the rat gastrocnemius muscle showed typical histological features at the time of death, including flat, peripherally located nuclei and parallel striated muscle fibers (Figure 3A). At 24 hours postmortem, skeletal muscle myofibers began to lose striation, and nuclei became eccentrically located (Figure 3B). By 48 hours, the gastrocnemius muscle showed eccentric pyknotic nuclei, widely spaced myofibers, and wavy nuclei (Figure 3C). At 72 hours postmortem, segmented muscle fibers, loss of intracellular nuclei, and the disappearance of cross striations were evident (Figure 3D). By 96 hours, the skeletal muscle tissue exhibited dilated, clogged blood vessels, fragmented muscle fibers with irregular nuclei, and large regions of tissue loss replaced by thin collagenous connective tissue (Figure 3E). A quantified comparison of muscle autolysis across different time intervals is shown in Table 3.

**Table 3:** Quantified comparison of muscle autolysis across postmortem intervals in rat groups

Group	0h	24h	48h	72h	96h	*P* value
**Muscle autolysis score mean**	**0.20**	**1.30**	**2.30^a^**	**3.20^ab^**	**3.90^ab^**	**<0.0001**
**SD**	**± 0.42**	**± 0.67**	**± 0.48**	**± 0.63**	**± 0.32**	

Values expressed as mean ± SD. Used test: Friedman test followed by post hoc Dunn's multiple comparisons test. a: Significance vs. 0h, b: Significance vs. 24h at *P* < 0.05.

## Discussion

In any postmortem investigation, one of the most crucial medico-legal questions is determining the time since death, also known as the postmortem interval. PMI refers to the period that has passed following a person's death. If the exact time of death is unknown, various medical and scientific methods are employed to estimate it, including examining blood markers, organs, and bodily fluids after death [16].

It is challenging to evaluate our results because studies have been done using various analytical techniques to determine the time of death, as evidenced by technological advancements. These studies measured different parameters to determine the PMI. The time of death has been predicted using a variety of biochemical, immunohistochemical, and histopathological approaches; however, the usefulness of these approaches is restricted. The current study found that the rats' bodies developed changes in serum HMGB1 levels after death. This research sought to determine whether serum HMGB1 could be used to estimate PMI. Our results revealed that HMGB1 levels increased up to 48 hours after death. This increase was most pronounced at 22 ± 2°C between zero and 48 hours. After peaking at 48 hours, the serum HMGB1 levels decreased over the following days at 72 and 96 hours. Measuring HMGB1 levels to assess the PMI is helpful because organs do not need to be homogenized; only serum samples are needed. ELISA allows for HMGB1 analysis within two days, making it a practical tool for PMI calculation. This approach may provide insights into the biochemical changes after death and offer potential markers for PMI estimation.

Our findings align with Kikuchi’s study on Wistar rats, which also demonstrated a time-dependent increase in HMGB1 levels during the first two days postmortem, with a peak on the third day followed by a decline on the fourth, then plateauing at a specific temperature [17]. Similarly, El-Din *et al*. highlighted the critical role of HMGB1 in PMI estimation within the timeframes examined in our study [18].

In addition, our research revealed a relationship between PMI and the degradation of the desmin protein. Desmin expression, as observed through immunohistochemical analysis, decreased from strong immunoreactivity in nearly all muscle fibers at 0 hours (38.19%) to minimal expression (1.09%) at 96 hours. Desmin is highly sensitive to the activities of calpains, proteasomes, and lysosomes, which are crucial for skeletal muscle cytoskeletal integrity [19]. Additionally, desmin makes up most of the intermediate filament that surrounds the sarcomere's Z-disk and connects it to the subsarcolemmal cytoskeleton, accounting for 0.35% of all proteins found in muscles [20].

Our results are consistent with earlier studies on pigs, which showed that desmin protein persists for a few days after death (up to 224 hours postmortem) [21]. Furthermore, our findings agree with Koohmaraie *et al*. [22], Taylor *et al*. [23], and Pittner *et al*. [24], all of whom documented the degradation of desmin as a reliable indicator of postmortem protein breakdown. Additionally, the products of desmin degradation in humans are comparable to those discovered in research examining the molecular makeup of muscle proteins in a variety of domestic animals, including cattle [25], pigs [26], and lambs [27], which showed regular degradation one to two days after death.

Moreover, our study's histological examination of muscle tissues revealed a strong correlation with PMI. Morphological changes in the gastrocnemius muscle could serve as valuable indicators for estimating the time of death. At 0 hours postmortem, the gastrocnemius muscle exhibited normal histological morphology, with parallel striated muscle fibers and flat, peripherally located nuclei. By 24 hours, changes in muscle striations and nuclear alterations were noted. After 48 hours, the muscle displayed cytoplasmic vacuoles and increased nuclear alterations. By 72 and 96 hours, the muscle showed a reduction in striations and nuclear presence, with fragmented muscle fibers and pyknotic nuclei.

These findings agree with Guerrero-Urbina *et al*. [28], who observed pyknotic nuclei in myofibers at 96-120 hours PMI and were not present at earlier postmortem intervals. The changes in the histology of the human lingual striated muscle make it possible to estimate PMI. Similarly, Yahia *et al*. [29] demonstrated that histopathological changes in the kidneys, liver, heart, and skeletal muscles could be used to estimate PMI in dogs. The histological changes observed in multiple studies, including our muscle histology results, support the use of histological examination as a reliable tool for PMI estimation. Our findings also align with Mostafa *et al*. [30], who found a strong correlation between histological changes in muscle tissue and postmortem intervals.

### Limitations

Postmortem, the gastrocnemius muscle is more stable compared with other bodily tissues. This study has shown that it is easily applicable to PMI investigations. To validate these results, more work is necessary. To assess future chosen biomarkers, a larger cohort of experimental animals would need to undergo targeted analysis in the following phase. A model for estimating PMI using muscle tissue might be developed if the concentrations of certain targeting minerals and multiple proteins were quantified. This would enable statistical analysis of the data. Applying techniques related to molecular levels to muscle will also be crucial.

## Conclusion

This study concluded that postmortem changes occur in a time-dependent manner, with a gradual deterioration of the gastrocnemius muscle’s histological structure as time progresses after death. The findings demonstrated a clear correlation between the PMI and the decline in the HMGB1 biomarker, along with changes in immunoreactivity and histopathological alterations in the muscle tissue. Ultimately, it was determined that the HMGB1 biomarker desmin expression and the observed histological changes in the gastrocnemius muscle could be reliable indicators for accurate PMI estimation. Both HMGB1 and desmin were identified as potential functional biomarkers for PMI in this study, which also emphasizes the need for further in-depth research in this area. The study suggests that these markers hold promise as valuable tools for postmortem analysis in humans and should be explored further in future investigations.

## Data Availability

The data supporting the findings of this study are available from the corresponding author upon reasonable request.

## Personal thanks

I would like to extend my sincere gratitude to Dr. Osama A. Elkashty, Assistant Professor of Oral Pathology at the Faculty of Dentistry, Mansoura University, for his invaluable assistance with the histopathological aspects of this research.
